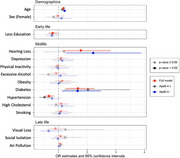# Life Course Modifiable Risk Factors of Dementia: Replicating the 2024 Lancet Commission Model in a Single Longitudinal Cohort

**DOI:** 10.1002/alz70860_107692

**Published:** 2025-12-23

**Authors:** Victoria J. Williams, Ralph Trane, Kamil Sicinski, Carol Roan, Pamela Herd, Michal Engelman, Sanjay Asthana

**Affiliations:** ^1^ University of Wisconsin‐Madison, Madison, WI, USA; ^2^ University of Wisconsin Madison, Madison, WI, USA; ^3^ Univeristy of Wisconsin ‐ Madison, Madison, WI, USA; ^4^ University of Michigan, Ann Arbor, MI, USA; ^5^ Wisconsin Alzheimer's Disease Research Center, University of Wisconsin School of Medicine and Public Health, Madison, WI, USA

## Abstract

**Background:**

Findings of the 2024 Lancet Commission suggest nearly 40% of dementia prevalence could be reduced by eliminating a set of 14 modifiable risk factors. Although promising, effect estimates derived from meta‐analytic approaches can be influenced by publication bias, inconsistently defined constructs/covariates, neglecting genetic interactions, and largely based on cross‐sectional findings. How the Lancet model would perform in predicting dementia within a single cohort, or vary based on genetic risk, remains unknown.

**Method:**

Leveraging data from 5,526 participants in the Wisconsin Longitudinal Study (WLS) with known dementia status, we aimed to replicate the Lancet model in a single population‐based longitudinal cohort. Risk factors were defined by Lancet suggested cut‐points and timing criteria, drawing from 70 years of prospectively collected WLS data. Unadjusted univariate analyses first evaluated independent associations between each risk factor and dementia status. We next used logistic regression with multiple imputations to model dementia outcomes by all risk variables simultaneously (controlling for age and sex), which was further stratified by ApoE‐4 carrier status.

**Result:**

When risk factors were evaluated independently, we found less early‐life education, mid‐life hearing loss and diabetes, and late‐life social isolation and visual loss associated with increased odds for dementia. Conversely, mid‐life hypertension associated with decreased dementia odds. Null effects (*p*>0.05) were observed for the remaining mid‐life (depression, physical inactivity, excessive alcohol, obesity, high cholesterol, smoking) and late‐life (social isolation, air pollution) predictors. When replicating the full Lancet model using logistic regression, only the effects of hearing loss, diabetes, and hypertension remained statistically significant. Stratified analysis among APoE‐4 carriers found that diabetes increased risk, whereas hypertension reduced risk for dementia. Among ApoE‐4 non‐carriers, only mid‐life hearing loss and diabetes associated with increased dementia odds.

**Conclusion:**

Replication of the 2024 Lancet model in a single lifecourse longitudinal cohort revealed only a restricted subset of modifiable risk factors that associated with dementia odds, raising concern for publication bias potentially inflating risk estimates in meta‐analytic approaches. History of mid‐life hypertension consistently associated with reduced dementia risk, contrary to model predictions. Divergent risk profiles by ApoE‐4 status highlight the importance of considering gene‐by‐environment interactions when formulating dementia risk models.